# Drug-Free Remission of VEXAS Syndrome Without Stem Cell Transplantation

**DOI:** 10.7759/cureus.109208

**Published:** 2026-05-19

**Authors:** Jenny Warren, Christina Smylie, Ardyth Milne

**Affiliations:** 1 Department of Medicine, Queen's University, Kingston, CAN; 2 Department of Medicine, University of Saskatchewan, Regina, CAN

**Keywords:** allele frequency, autoinflammatory, myelosuppression, uba1, vexas

## Abstract

Vacuoles, E1 enzyme, X-linked, autoinflammatory, somatic (VEXAS) syndrome is a recently discovered acquired autoinflammatory disease caused by a point mutation in the *UBA1* gene of myeloid progenitor cells. We present a case of an 81-year-old male with VEXAS syndrome. His manifestations included severe cutaneous symptoms, cytopenias, and other organ inflammation, which initially posed a significant diagnostic challenge, given that VEXAS syndrome had only recently been discovered at that time. Multiple therapies were trialed, including courses of antibiotics and immunomodulatory agents. The diagnosis was ultimately confirmed by bone marrow biopsy and genetic testing, with a positive UBA1 mutation with an allele frequency of 10% in the myeloid progenitor cell line. Symptom control was ultimately achieved with methotrexate and chronic prednisone at low-to-moderate doses. Unfortunately, he developed methotrexate-induced bone marrow toxicity, identified during a hospitalization for community-acquired pneumonia; thus, the methotrexate was discontinued. Shortly thereafter, he achieved not only resolution of his autoinflammatory symptoms and hematologic abnormalities, but also clearance of the *UBA1 *gene mutation. He has since tapered off prednisone entirely and remains in drug-free biochemical and symptomatic remission. This case raises questions about the possible role of higher-intensity initial treatment in the management of VEXAS syndrome to target the bone marrow. Such therapies could include agents that induce temporary myelosuppression with the goal of subsequent treatment-free remission. Furthermore, we hypothesize that his relatively low UBA1 allele frequency may serve as a diagnostic marker for an increased likelihood of remission.

## Introduction

Autoinflammatory diseases are rare disorders characterized by overactivation of the innate immune system. They are typically genetic disorders that arise in childhood. Further studies have identified acquired genetic mutations leading to autoinflammatory disease in older adults. Vacuoles, E1 enzyme, X-linked, autoinflammatory, somatic (VEXAS) syndrome is a recently discovered acquired autoinflammatory disease. Since its original description by Beck et al. in 2020 [1], VEXAS syndrome has proven challenging to diagnose and treat due to its varied phenotypic presentations of inflammation [2-5]. VEXAS syndrome is characterized by adult-onset autoinflammatory symptoms developing in a variety of organs, including cutaneous, renal, pulmonary, and musculoskeletal systems. VEXAS syndrome also presents with fevers, cytopenias, myeloid dysplasia, characteristic bone marrow vacuolization, and is defined by diagnostic X-linked mutations in the *UBA1* gene [1]. Given the UBA1 mutations are acquired and X-linked, the majority of patients are male and present later in life. 

There is significant morbidity and mortality associated with this condition secondary to uncontrolled severe systemic inflammation, symptomatic cytopenias, and infections in the setting of immunosuppressive therapies. Given its relative novelty, effective long-term treatment modalities are still being investigated. Thus far, promising treatment strategies include various immunosuppressants, hypomethylating agents, and hematopoietic stem cell transplantation (HSCT) in appropriate candidates [6-8]. Currently, the only established curative therapy is HSCT. However, given that VEXAS syndrome typically affects older adults, this is often not a realistic treatment option for many patients due to the high degree of short-term and long-term toxicities from HSCT. Most therapies currently being used to manage VEXAS syndrome involve long-term immunomodulatory agents with a goal of controlling symptoms and cytopenias to minimize the use of chronic glucocorticoids or transfusion dependence [9]. 

The presence of the UBA1 mutation in the peripheral blood, or more simply the allele frequency, has been shown to vary significantly in patients diagnosed with VEXAS syndrome. In the initial identification of VEXAS syndrome, there was a reported allele frequency of >71% in affected individuals, with unaffected general population undergoing Sanger sequencing having no more than a 5% variant allele frequency [1]. Further studies have identified a wide range of allele frequencies among patients with VEXAS syndrome [10-13]. Some studies have described a correlation between allele frequency and burden of disease, with the presence of UBA1 mutations sometimes preceding symptoms [10]. A wide spread of UBA1 allele frequencies have been identified in patients with VEXAS syndrome, with the pattern trending towards higher UBA1 frequency associated with more severe disease. 

This case describes the rarity of a patient with VEXAS syndrome achieving drug-free remission without the use of HSCT. We hypothesize there may be a role for alternative treatment strategies using higher-intensity initial treatment, with the goal of inducing treatment-free remission. Our case also raises further questions about the influence of allele frequencies on disease intensity and remission possibilities. The significance of the high degree of variability in UBA1 mutation allele frequency is still under investigation; however, there may be evidence to suggest it may trend with disease severity.

An initial case description of this patient was previously presented as a poster at the CRA Annual Meeting 2004, Winnipeg, Canada [14].

## Case presentation

**Figure 1:**
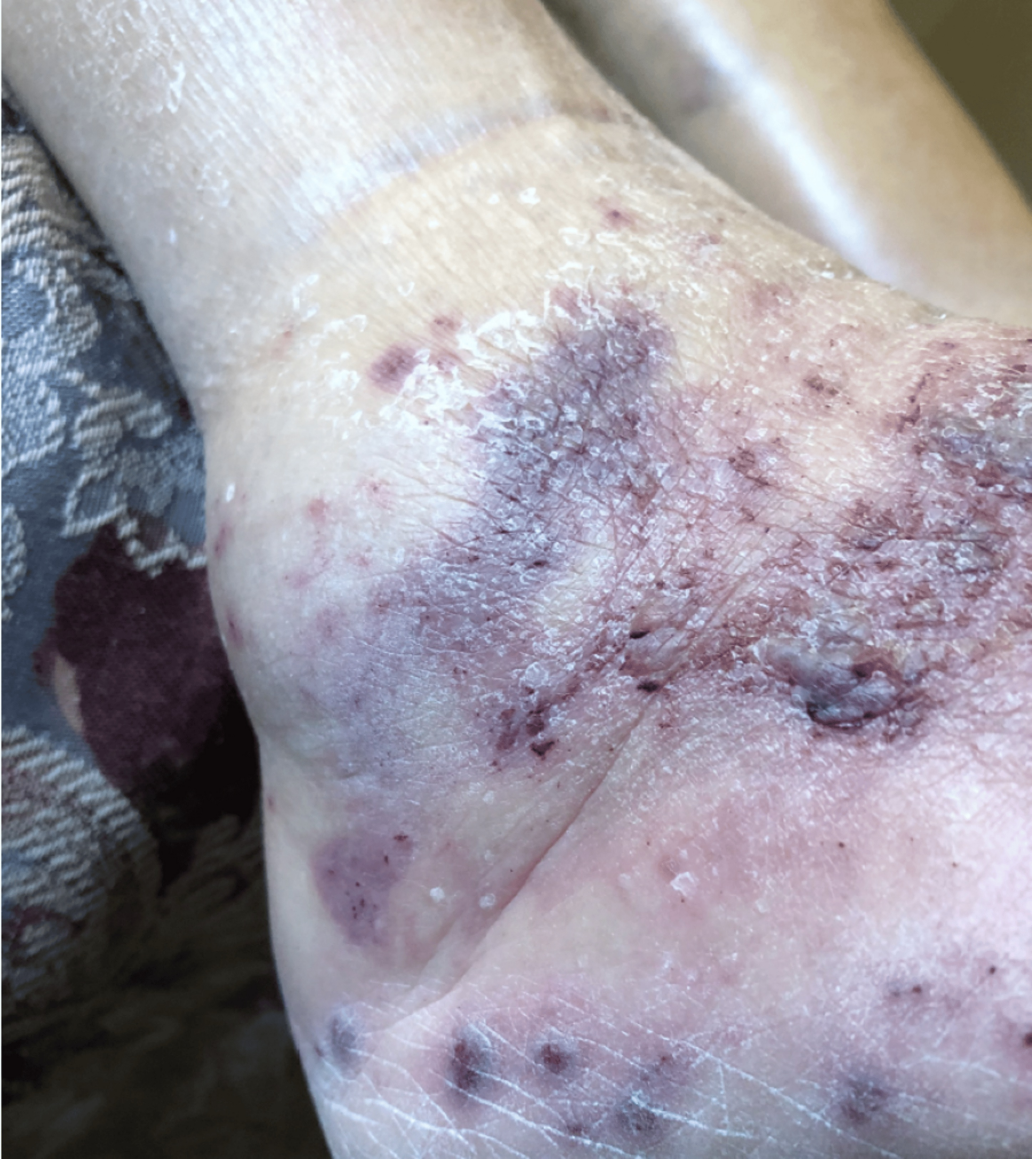
Leukocytoclastic vasculitis as a manifestation of VEXAS syndrome. The dorsum of the foot and lateral hindfoot with palpable purpura is shown. A skin biopsy revealed leukocytoclastic vasculitis. This rash was among the many cutaneous symptoms observed in the patient's presentation of VEXAS syndrome. VEXAS: Vacuoles, E1 enzyme, X-linked, autoinflammatory, somatic.

A previously healthy 81-year-old man presented in 2021 with multiple relapsing symptoms, including biopsy-proven cutaneous vasculitis, arthritis, superficial migratory thrombophlebitis, severe peri-orbital and facial swelling, uveitis, and fleeting pulmonary infiltrates. The most severe of his manifestations were cutaneous in nature. However, the rashes he experienced were often transient, which led to difficulty in timely performance of a skin biopsy. With close monitoring and frequent patient interaction, a skin biopsy of palpable purpura was eventually obtained, with a finding of leukocytoclastic vasculitis (Figure 1). 

Blood work abnormalities included elevated ESR, CRP, persistent lymphopenia, and macrocytic anemia. Given the non-specific nature of his symptoms, many differential diagnoses were explored. At one point, he was thought to have Trousseau syndrome, a paraneoplastic condition that can cause superficial thrombophlebitis in the setting of pancreatic cancer. He was thoroughly investigated for underlying malignancy, including performance of a colonoscopy; computed tomography (CT) imaging of the chest, abdomen, and pelvis; magnetic resonance cholangiopancreatography (MRCP); cystoscopy; and evaluation of serum markers. All malignancy workup results returned negative.

**Figure 2:**
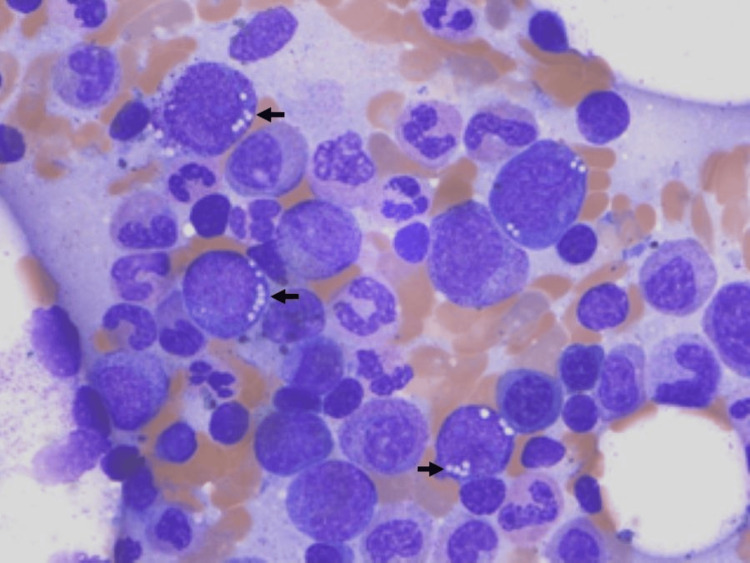
Bone marrow biopsy diagnostic of VEXAS syndrome. The initial diagnostic bone marrow biopsy showing cytoplasmic vacuoles in early myeloid and erythroid cells, highlighted by black arrows. This finding greatly increased suspicion for VEXAS syndrome. Confirmation of the diagnosis of VEXAS syndrome was then achieved through genetic testing of the peripheral blood. VEXAS: vacuoles, E1 enzyme, X-linked, autoinflammatory, somatic.

Given the inflammatory symptoms concerning for infection, he was initially treated empirically with antibiotics without significant improvement. Infectious causes were also thoroughly explored, with negative bacterial cultures, viral cultures, hepatitis, Lyme, syphilis, and viral serology. Once infection and malignancy were ruled out through comprehensive investigations, he was managed with conventional disease-modifying antirheumatic drugs (DMARDs) and corticosteroids given the wide differential diagnosis of underlying inflammatory causes. He showed a significant response to prednisone. Other diagnoses that were explored included acquired C1 esterase deficiency given his facial swelling, sarcoidosis, celiac disease, systemic lupus erythematosus, myositis, and IgG4 disease. These considerations also did not yield a diagnosis. Through multidisciplinary discussions, the possibility of VEXAS syndrome arose when considering his cutaneous manifestations, macrocytic anemia, and systemic inflammation. Ultimately, after bone marrow biopsy showed characteristic vacuoles and genetic testing documented the UBA1 gene mutation, with an allele frequency of 10% in the myeloid lineage of peripheral blood, a diagnosis of VEXAS syndrome was made (Figure 2).

He achieved symptomatic relief of his cutaneous flares on methotrexate at 15 mg weekly. He was also longitudinally maintained on low-dose prednisone, with doses varying between 5 and 15 mg per day, trying to maintain the minimum prednisone possible to control symptoms. He did try hydroxychloroquine for a period, without significant improvement. At one point, methotrexate was discontinued due to unclear benefit, but he developed a significant cutaneous flare within three months. The methotrexate was restarted and subsequently increased to 25 mg weekly after he had a subsequent episode of nasal chondritis. 

**Figure 3:**
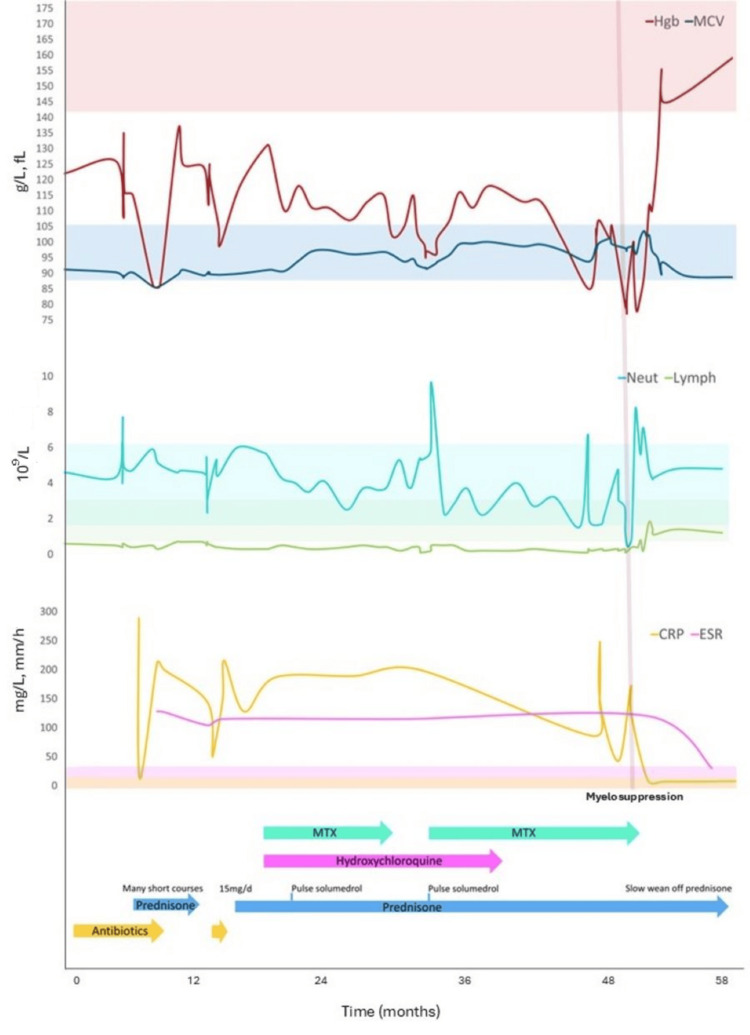
Timeline of patient’s bloodwork and provided treatments. Graph of bloodwork trend over time of hemoglobin (Hgb, g/L), mean corpuscular volume (MCV, fL), neutrophil count (neut., x10^9^/L), lymphocytes (lymph., x10^9^/L), erythrocyte sedimentation rate (ESR, mm/h), and C-reactive protein (CRP, mg/L). The coloured bands correspond to the normal ranges. Also shown are the treatments provided for his VEXAS syndrome, as they were started and stopped over time. Prednisone was given in ranges between 5 and 15 mg, following showing symptomatic response to 15 mg daily. The vertical transparent red band marks a shift in his bloodwork showing myelosuppression evidenced by pancytopenia, with a subsequent resolution of his Hgb, MCV, neutrophils, lymphocytes, ESR, and CRP. His methotrexate was discontinued at the time of myelosuppression, and shortly thereafter, prednisone was weaned off from 7.5 mg daily without any worsening of his bloodwork values. Since then, his complete blood count and inflammatory markers have remained within normal limits. The figure was created using Microsoft Excel and assembled in Microsoft PowerPoint. MTX: methotrexate.

Approximately nine months after the methotrexate dose increase, he was hospitalized for pneumonia. During this presentation, he was also found to have pancytopenia, with a hemoglobin of 78 g/L, platelets of 55 × 10^9^/L, neutrophils of 0.4 × 10^9^/L, and a decreased reticulocyte count, consistent with probable myelosuppression from methotrexate. His methotrexate was discontinued, while he remained on prednisone 7.5 mg daily. Over the following six to eight weeks, his hematologic abnormalities and inflammatory markers normalized without the need for transfusion, representing the first time his bloodwork had normalized in the previous four years since initial presentation. Although he did not undergo a bone marrow biopsy in the hospital to investigate his pancytopenia, the removal of the methotrexate was sufficient for bone marrow recovery. After it was established that he was in clinical and hematologic remission, repeat genetic testing of the peripheral blood showed that he was now negative for the UBA1 mutation. He was subsequently tapered off oral corticosteroids and has remained in drug-free remission from autoinflammatory symptoms in the years thereafter (Figure 3).

## Discussion

This case postulates the role of alternative treatment approaches for VEXAS syndrome, as well as potential prognostication strategies. This patient had a relatively low UBA1 mutation allele frequency of 10% on initial testing but was nonetheless fairly symptomatic, requiring chronic corticosteroids for symptom control. In our case, once the patient achieved clinical remission, there was no detectable UBA1 mutation, supporting the hypothesis that the UBA1 mutation burden may correlate with symptomatology [11].

A recent case report by Sockel et al. described a patient with VEXAS syndrome with concurrent MDS who achieved a treatment-free remission and clearance of the UBA1 mutation after 44 months of treatment with azacitidine [13]. Similar findings were identified in a recent review from FRENVEX (French VEXAS study group), in which patients with VEXAS syndrome who achieved a molecular response to azacitidine, defined in the paper as a ≥25% relative reduction in UBA1 variant allele frequency, all achieved clinical and hematologic responses to treatment. Of these patients who achieved both clinical and molecular responses, a small number discontinued therapy and were able to maintain treatment-free remission for a median of about three years before relapse [15]. While the treatment modalities differ from the case presented here, a similar finding of suppression of the UBA1 mutation correlating with clinical response to therapy is further supportive of the idea that UBA1 monitoring may be of clinical utility in the long-term management of VEXAS syndrome.

We query whether UBA1 mutation allele frequencies may help guide possible therapies or prognostication in VEXAS syndrome, in addition to being a possible marker of disease activity. Given this patient’s prolonged clinical and biochemical remission after sustaining temporary myelosuppression, the low initial allele frequency may have possibly been a favourable prognostic indicator. It is worth noting, however, that his initial UBA1 mutation testing was done after he had already been started on immunosuppressive therapy, so his true peak variant allele frequency may have in fact been greater than 10%. Further studies evaluating variation in allele frequencies and phenotypic presentations should be performed to assess the prognostic value of the UBA1 variant allele frequency. Additionally, further studies to assess the response to therapy by trending not only clinical features but also genetic biomarkers with the UBA1 mutation may help guide future therapies for VEXAS syndrome, particularly in patients for whom achieving treatment-free remission is considered achievable. Serial monitoring of the UBA1 mutation during treatment and after remission is achieved may be of clinical utility as a possible predictor of relapse.

We question if future treatments for VEXAS syndrome may involve higher intensity treatments upfront to achieve a more robust clinical and biochemical remission, possibly followed by a treatment-free period. Currently, the only available curative treatment is HSCT, which is associated with significant short- and long-term toxicity and is not always feasible in older patients, who represent the majority of the population with VEXAS syndrome. Further investigation into medications such as high-dose methotrexate, either to induce temporary myelosuppression and possible eradication of the aberrant myeloid progenitor clone, may broaden our understanding of possible curative treatments for VEXAS syndrome. Expanding treatment options for VEXAS syndrome would prove valuable as an alternative option for patients who are too frail to undergo HSCT yet still seek either curative-intent treatment or treatment-free remission.

## Conclusions

This case of a patient who inadvertently achieved treatment-free remission of VEXAS syndrome after brief myelosuppression from methotrexate raises several questions for future studies. We theorize that the UBA1 allele frequency may be used as a biomarker to assess disease severity or possibly as a prognostic indicator for VEXAS syndrome. We furthermore suggest that serial monitoring of UBA1 variant allele frequency may be of clinical utility in the chronic management of VEXAS syndrome. Further study into the validity and utility of methotrexate or other therapies with myelosuppression intent may be valuable in patients seeking treatment-free disease remission as an alternative to HSCT or other chronic therapies. Given the secondary effects of long-term steroids, and the severity of HSCT, upfront high-intensity therapies could possibly serve as a preferable treatment approach. This case report reflects the emerging ideas for future therapeutic considerations in VEXAS syndrome. 
